# Structure-Property Relationships Governing Species Dependent Response in Alkali-Assisted Chemical-Mechanical Pulping of Hardwoods

**DOI:** 10.3390/polym18101195

**Published:** 2026-05-13

**Authors:** Yingjie Wang, Bin Wang, Peng Huang, Yan Wu, Fengshan Zhang, Zhiqiang Sun, Hongxia Ma, Wenguang Wei, Kefu Chen

**Affiliations:** 1 Plant Fiber Material Science Research Center, State Key Laboratory of Advanced Papermaking and Paper-Based Materials, School of Light Industry and Engineering, South China University of Technology, Guangzhou 510640, China; 2 Shandong Huatai Paper Co., Ltd., Dongying 257335, China; 3 State Key Laboratory of Green Papermaking and Resource Recycling, Qilu University of Technology (Shandong Academy of Sciences), Jinan 250353, China; 4 Ocean University of China, Qingdao 266100, China; 5 Guangdong Academy of Forestry, Guangzhou 510520, China

**Keywords:** alkali pretreatment, fiber morphology, lignin-carbohydrate complexes

## Abstract

The efficient utilization of hardwood lignocellulosic biomass has attracted increasing attention as a sustainable strategy for the high-value conversion of renewable resources. Chemical-mechanical pulping (CMP) is a promising route for hardwood utilization; however, its performance is strongly influenced by species-dependent differences in chemical composition, macromolecular structure, and physical accessibility. In this study, four representative hardwood species (poplar, sycamore, eucalyptus, and acacia) were selected as model feedstocks to investigate the relationships between structural characteristics and CMP performance in alkali-assisted systems. The chemical composition and structural features of cellulose, hemicellulose, lignin, and lignin-carbohydrate complexes were characterized, together with key physical parameters including density, porosity, and fiber morphology. The effects of alkali charge on fiber softening, fibrillation development, and paper properties were then evaluated. The results revealed pronounced species-dependent differences in alkali response, which were closely correlated with variations in cellulose supramolecular organization, hemicellulose substitution characteristics, lignin structural features, lignin-carbohydrate associations, and wood microstructure. This study provides a comprehensive qualitative comparative analysis of the relationships between wood structural features and CMP performance. Hardwoods with lower density and higher porosity exhibited more efficient alkali penetration and superior performance under mild conditions, whereas denser species such as sycamore and eucalyptus required higher alkali charge. This work provides important insights into the structure-performance relationships governing alkali-assisted CMP behavior, and offers useful guidance for the efficient utilization of lignocellulosic biomass in pulp and paper applications.

## 1. Introduction

Lignocellulosic biomass is an abundant renewable resource composed primarily of cellulose, hemicellulose, and lignin, which are organized into a complex hierarchical structure through physical entanglement and chemical association [1,2]. As a major class of natural polymeric materials, lignocellulosic biomass has attracted sustained interest in the pulp and paper field because the composition, structure, and interactions of its major macromolecular components directly determine processing behavior and end-use performance [3,4]. Among lignocellulosic resources, hardwoods are important raw materials for pulp production because of their wide availability, high fiber yield, and broad industrial relevance [5]. However, different hardwood species exhibit substantial variations in cellulose organization, hemicellulose substitution patterns, lignin structure, and lignin-carbohydrate associations, as well as in cell wall architecture and physical accessibility, which can lead to markedly different pulping responses [6,7,8]. Therefore, understanding the species-dependent structural characteristics of hardwood biomass is essential for the efficient utilization of these renewable resources in pulp and paper applications.

Chemical-mechanical pulping (CMP) is an important route for lignocellulosic biomass utilization because it combines chemical softening with mechanical fiber liberation, thereby promoting fiber separation while preserving a substantial fraction of the carbohydrate framework compared with more severe pulping processes [9,10]. In alkali-assisted CMP, sodium hydroxide pretreatment partially disrupts lignin-rich domains, weakens lignin-carbohydrate associations, and promotes swelling and softening of the fiber wall before refining [11]. The efficiency of this process largely depends on the chemical and supramolecular characteristics of cellulose, hemicellulose, lignin, and lignin-carbohydrate complexes (LCCs). Variations in cellulose crystallinity and degree of polymerization can affect resistance to swelling and mechanical damage, while differences in hemicellulose substitution patterns and alkali sensitivity influence matrix loosening and inter-fiber bonding [12]. Likewise, lignin aromatic structure, ether linkage abundance, and LCC features determine the accessibility and reactivity of the lignified cell wall under alkaline conditions [5]. As a result, the balance between fiber preservation, component dissolution, and fibrillation development during CMP is strongly species-dependent.

In addition to chemical composition and structure, the physical accessibility and microstructure of hardwoods also play decisive roles in alkali-assisted pulping. Parameters such as density, porosity, pore structure, cell wall thickness, lumen dimension, and fiber morphology directly affect liquid transport, chemical diffusion, and resistance to mechanical refining [13]. These anatomical and microstructural characteristics determine how efficiently alkaline liquor can penetrate the wood matrix and how effectively the cell wall can be softened before mechanical treatment [14,15]. Hardwoods with more compact structures, lower porosity, or less accessible cell walls often require stronger chemical action or higher refining intensity to achieve sufficient fiber liberation, whereas more porous and structurally accessible species respond effectively under milder conditions [16,17]. Previous studies have mostly focused on cellulose isolated from a single biomass source or on single structural characteristics such as chemical composition, crystallinity, degree of polymerization, or thermal stability. However, limited attention has been paid to systematic cross-species comparisons of wood-derived celluloses. In particular, the relationship between species-dependent chemical composition, multiscale cellulose structure, and processing-related thermal behavior remains insufficiently clarified. This lack of integrated understanding limits the rational selection of suitable woody raw materials for subsequent cellulose processing and high-value utilization.

To fill this research gap, this study conducted a comparative study on four representative types of timber (poplar, sycamore, eucalyptus, and acacia). This study focuses specifically on key structural descriptors related to alkali penetration, fiber softening efficiency, mechanical finishing resistance, and final paper properties. Examples include the supramolecular structure of cellulose, the characteristics of lignin-carbohydrate complexes, cell wall compactness, and pore structure. On this basis, the influence of alkali charge on fiber softening, fibrillation development, fiber quality, crystalline structure evolution, and paper strength properties was evaluated. This work provides an integrated qualitative understanding of the relationships between wood structure, alkali response, and CMP performance, and identifies key structural descriptors associated with species-dependent behavior.

## 2. Materials and Methods

### 2.1. Materials

The poplar, sycamore, eucalyptus, and acacia wood chips used in the experiment were purchased from Shandong Sun Paper Co., Ltd. (Jining, China). The wood chips were industrial materials made up of heartwood and sapwood, as well as longitudinal sections, and they were used as received after debarking. All samples were derived from approximately 5-year-old trees. Prior to use, the samples were air-dried under ambient laboratory conditions (room temperature, natural ventilation) and stored in sealed containers. All samples were treated under identical conditions. The chemical composition of the wood samples (Table 1) was determined according to the NREL Laboratory Analytical Procedures. Specifically, ash content and extractives content were measured following NREL/TP-510-42622 and NREL/TP-510-42619, respectively, while cellulose, hemicellulose, lignin, and alcohol-benzene extractives were analyzed based on a modified NREL/TP-510-42618 protocol. Water was obtained from ultrapure water prepared in the laboratory. All reagents used in experiments were of analytical grade and used without any further purification. Glacial acetic acid, sodium hypochlorite, benzene, ethanol, DMSO, and NaOH were purchased from Shanghai McLean Biochemical Technology Co., Ltd. (Shanghai, China).

### 2.2. Alkali-Assisted Chemical-Mechanical Pulping and Handsheet Preparation

One kilogram (oven-dry basis) of cleaned, air-dried raw material was first soaked in water at 25 °C for 24 h to ensure thorough impregnation. Subsequently, 2 wt% NaOH was introduced, and the material was subjected to steam-assisted alkaline pretreatment for 20 min. After steaming, the treated material was subjected to mechanical treatment using a twin-screw extruder (Model 120, Weifang, China). The extruder consisted of two working zones equipped with forward and conveying screw elements. Wood chips were continuously fed into the extruder at a screw speed of 140 rpm, with a processing capacity of approximately 2 t/h. During this process, the material experienced combined compressive, shear, and frictional forces, which promoted fiber separation and fibrillation. All extrusion treatments were conducted under identical operating conditions. The material was then air-dried, and its moisture content was determined for subsequent processing.

For alkali-assisted chemical-mechanical pulping, 100 g (oven-dry basis) of the squeezed and torn material was placed in a sealed reaction bag. Deionized water was added at a solid-to-liquid ratio of 1:4, together with NaOH at charges ranging from 4~8%. The mixture was reacted at 90 °C for 40 min, with manual kneading performed every 10 min to ensure uniform alkali penetration and reaction. After the alkaline treatment, the material was subjected to two-stage mechanical refinement using a high-consistency hot refiner, with plate gaps set at 0.5 mm and 0.2 mm, respectively.

The resulting pulp was then desized at 80 °C for 40 min to remove residual surface contaminants and extractives. After desizing, the pulp was screened using a flat screen classifier (S401700001, Munich, Germany) equipped with a 0.25 mm slot screen to obtain the accepted fraction, which was designated as the accepted pulp. Subsequently, 30 g (oven-dry basis) of the screened pulp was diluted to a consistency of 10% and further refined using a PFI mill to a target beating degree of 40 ± 2 °SR. Handsheets with a basis weight of 80 g/m^2^ were prepared from the refined pulp, and the physical properties of the resulting paper were systematically evaluated.

### 2.3. Chemical Composition and Component Separation

#### 2.3.1. Monosaccharide Composition Analysis

A total of 0.03 g of wood powder (oven-dry basis) was added to 0.3 mL of 72% H_2_SO_4_, which was stirred intermittently with a glass rod and kept warm at 30 °C for 60 min, before 8.4 mL of water was added after the end of the insulation. The digestion tube is placed in a multifunctional intelligent digestion apparatus (GL-16, Guangzhou, China) at 121 °C for 60 min. The supernatant was obtained after filtration through a G3 filter [18].

Monosaccharide contents were measured with a high-performance liquid chromatography system (HPLC, Agilent 1260 infinity, Waltham, MA, USA) equipped with a Bio-Rad Aminex HPX-87H column (300 × 7.8 mm) and refractive index detector (Agilent 1260 II, Waltham, MA, USA). The column temperature was set at 60 °C, and 5 mmol/L aqueous sulfuric acid solution was used as the mobile phase at a flow rate of 0.6 mL/min. The monosaccharide content was determined using ion chromatography (Thermo Scientific ICS-5000+, Waltham, MA, USA) equipped with an anion-exchange column (CarboPacPA-20, 150 × 3 mm).

The monosaccharide hydrolysis rate was calculated relative to the original bark mass according to Equation (1):

(1) $$R(\%)=\frac{c_{m}\times v_{b}}{m_{b}}\times 100$$

where *R* is the monosaccharide hydrolysis rate (%), *c_m_* is the monosaccharide concentration (g/L), *v_b_* is the monosaccharide solution volume (L), and *m_b_* is the initial mass of WIB (g).

#### 2.3.2. Separation of Cellulose, Hemicellulose, Lignin, and LCCs

After benzene-ethanol extraction, holocellulose was isolated from the wood powder using an acidified sodium chlorite method [19]. CH_3_COOH and NaClO_2_ were added to the extracted wood powder, and the reaction was carried out at 70 °C until complete delignification was achieved. The obtained holocellulose was subsequently treated with NaOH at 25 °C under a nitrogen atmosphere to separate cellulose and hemicellulose. After filtration, the solid residue was collected as cellulose, while hemicellulose was recovered from the filtrate. Lignin was isolated from the benzene-ethanol extracted wood powder using a 1,4-dioxane/water (9:1, *v*:*v*) solvent system. The wood powder was first subjected to ball milling to enhance lignin dissolution, followed by extraction with the dioxane solution. The resulting crude lignin solution was concentrated and precipitated by dropwise addition into acidified deionized water. The precipitate was collected, washed, and dried for further analysis.

LCCs were prepared following a modified dissolution-precipitation procedure [20]. Specifically, 20 g of the purified material was treated with a 96% 1,4-dioxane solution at a solid-to-liquid ratio of 1:20 under light-protected conditions and heated for 24 h. After washing and vacuum drying at 50 °C, the solid was dissolved in acidified aqueous solution to remove insoluble residues. The LCC fraction was subsequently precipitated by dropwise addition of deionized water, collected from the supernatant, and recovered by cooling and drying.

### 2.4. Structural Characterization

#### 2.4.1. Fiber Crystallinity Analysis (XRD)

We cut a piece of paper to an appropriate size and laid it flat on the amorphous glass sample stage, before placing it on the D8-ADVANCE X-ray diffractometer for testing [21]. The radiation source was a copper target X-ray tube (λ = 1.54184 nm), the sample test diffraction angle was 10~40°, the scanning speed was 20 °/min, the working voltage was 40 kV, and the working current was 40 mA. The crystallinity (*X_c_*) is calculated according to the following formula:

(2) $$\mathrm{Crystallinity}\;X_{c}=\frac{I_{002}-I_{am}}{I_{002}}\times 100\%$$

where *I*_002_ is the crystal plane diffraction intensity (002), *I_am_* is the diffraction intensity of the amorphous region, and *I_am_* of cellulose I is the diffraction intensity at 2θ = 18.0°.

#### 2.4.2. Degree of Polymerization (DP) Measurement

The degree of polymerization (DP) of cellulose was determined by the viscometric method using cupriethylenediamine (CED) solution. Specifically, 0.2 g of cellulose was added to a 30 mL brown bottle together with five copper strips. Subsequently, 30 mL of 0.5 M CED solution was added, along with glass beads to facilitate air removal. The bottle was sealed and shaken for 15 min to ensure complete dissolution of the cellulose, followed by incubation in a water bath at 25 °C for 30 min.

The efflux time (*t_n_*) of the solution was measured using a Cannon-Fenske capillary viscometer. The relative viscosity (*η_relative_*) was calculated according to Equation (3):

(3) $$\eta_{relative}=h_{n}\times t_{n}$$

where *h_n_* is the viscometer constant (s^−1^), and *t_n_* is the sample outflow time (s).

The intrinsic viscosity [*η*] was calculated using the Mark-Houwink-Sakurada equation. Finally, the degree of polymerization was calculated using Equation (4):

(4) $${DP}^{0.905}=0.75[\eta ]$$

#### 2.4.3. Scanning Electron Microscopy (SEM)

The wood chips were soaked in water at room temperature to remove the air from the wood chips. The slice samples (50 μm) were cut by cryo-microtome at −30 °C.

The SEM image of the outer surface of the samples was obtained using a scanning electron microscope (JEM 2100F, Tokyo, Japan) at an accelerating voltage of 15 kV. Before imaging, we used conductive glue to directly stick the dried sample on the sample block, and we also used a small ion sputtering instrument (ETD2000, Guangzhou, China) to plate gold for 300 s.

#### 2.4.4. FT-IR, TGA, and 2D-HSQC NMR Analysis

The sample was moistened evenly with a small amount of water, pressed into small round tablets using a tablet press, and then dried for later use. FT-IR spectra were acquired by the use of a Fourier infrared diffractometer (Vertex 70, Munich, Germany). The test used a resolution of 4 cm^−1^, a scan time of 32 scans, and a wavelength range of 500–4000 cm^−1^. TGA was tested using a thermogravimetric analyzer (TG209F3, Munich, Germany). We took about 5~8 mg of sample and heated it from 40 °C to 800 °C at a heating rate of 10 °C/min under a nitrogen atmosphere. The 2D-HSQC NMR was carried out on a Bruker AVANCE NEO 500 M spectrometer (Karlsruhe, Germany). Specific details are as follows: 50 mg of sample was dissolved in dimethyl sulfoxide-d6, and the dissolved sample was added to an NMR tube. The sample was tested under NMR for 8–10 h.

### 2.5. Physical, Morphological, and Paper Property Evaluation

Wood porosity and pore diameter were assessed via a high-performance fully automated mercury porosimeter (AutoPore Iv 9510, Norcross, GA, USA). Nanoindentation technology was used to replace the dynamic mechanical analyzer (DMA) to measure the hardness of fibers (Bruker Hysitron TI Premier, Karlsruhe, Germany). Fiber quality analysis was performed using the Morfi Compact fiber analyzer. The physical properties of the paper were tested using an LW thickness gauge, tensile strength tester, tear strength tester, and bursting strength tester. All experiments were conducted in triplicate unless otherwise stated, and the results are expressed as mean values ± standard deviation (SD).

## 3. Results and Discussion

### 3.1. Chemical Integrity of Hardwood Components

**Figure 1:**
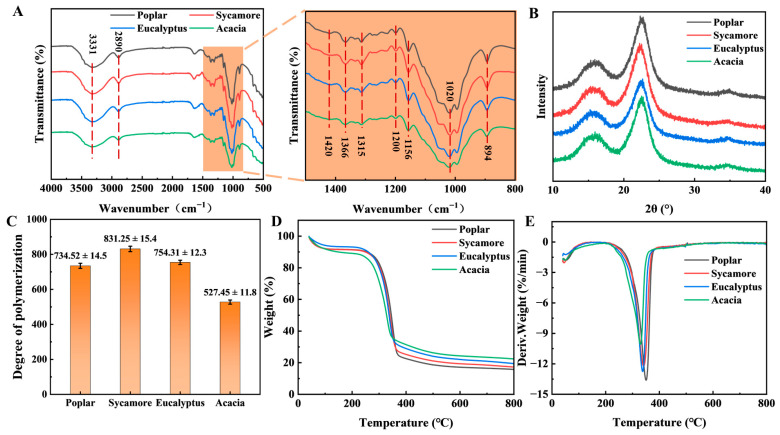
Structural and thermal analysis of cellulose isolated from four hardwood species. (**A**) FT-IR spectra, (**B**) X-ray diffraction patterns, (**C**) degree of polymerization, (**D**) TG, and (**E**) DTG curves.

Cellulose isolated from four hardwood species was subjected to FT-IR, XRD, DP, and thermogravimetric analysis. Figure 1A presents the FT-IR spectra of celluloses derived from the four hardwood species. The absorption peak at 3331 cm^−1^ originates from the stretching vibration of the O-H bonds in cellulose. This reflects intramolecular and intermolecular hydrogen bonds in cellulose [22]. The basic backbone structure of the four hardwood celluloses is well preserved. A stronger absorption peak indicates a more compact molecular chain arrangement within the crystalline region of cellulose, with a more stable hydrogen bond network between hydroxyl groups. The absorption peak at 2890 cm^−1^ corresponds to C-H stretching vibrations of methyl and methylene groups. The absorption peak at 1610 cm^−1^ is attributed to the bending vibration of absorbed water within the cellulose matrix. Characteristic cellulose bands were observed at 1420 cm^−1^ (CH_2_ symmetric bending), 1366 cm^−1^ (C-H bending), and 1315 cm^−1^ (C-C and O-H bending vibrations). The absorption peak at 1420 cm^−1^ reflects the crystalline cellulose region. The absorption peak at 1366 cm^−1^ reflects the regular arrangement of cellulose chains and the state of hydrogen bonds. Stronger absorption intensity indicates a more regular arrangement of cellulose chains and higher crystallinity. The higher intensity of the absorption peak at this point in sycamore indicates a more stable cellulose structure, a more ordered crystalline structure, and higher crystallinity. The peak at approximately 1200 cm^−1^ is associated with C-O stretching, while the band at 1156 cm^−1^ corresponds to asymmetric stretching of the C-O-C linkage in cellulose, reflecting the presence of glycosidic bonds and alcoholic functional groups. Notably, the relatively higher intensity of this band in sycamore-derived cellulose suggests a greater abundance of accessible hydroxyl groups, which enhances alkali swelling and fiber softening during CMP processing. The absorption peak at 1020 cm^−1^ comes from the vibration of the C-O-C pyran ring skeleton in cellulose, indicating the vibration of the glycosidic bond and alcohol group in the cellulose molecule. The absorption peak at 894 cm^−1^ is characteristic of the β-1,4-glycosidic bond in cellulose. The predominant cellulose peak in this region is poplar, followed by sycamore, eucalyptus, and acacia. These findings indicate potential variations in crystalline domain exposure and glycosidic bond accessibility among the four hardwood celluloses. The absorption peaks of sycamore at 3331, 1420, 1366, 1156, and 1120 cm^−1^ show relatively high intensities and concentrated peak shapes. This indicates that its cellulose skeleton is intact, with a relatively regular chain arrangement. The structure is relatively stable, the crystalline structure is relatively ordered, and the crystallinity is high. In contrast, the absorption peaks of acacia show relatively low intensities and broader peak shapes. This indicates that the cellulose structure of acacia is relatively unstable, and the crystallinity is relatively low.

### 3.2. Crystalline Organization, Molecular Weight, and Thermal Stability of Cellulose

Figure 1B shows the XRD patterns of celluloses extracted from four hardwood species. All samples exhibit the characteristic diffraction peaks of cellulose I, with the most intense reflection observed at the (002) crystal plane. The diffraction intensity at the crystal plane I_002_ is highest for sycamore cellulose. The crystallinity of cellulose, from highest to lowest, is sycamore, eucalyptus, poplar, and acacia. The crystallinity values are 72.16, 70.95, 70.16, and 67.48, respectively. Notably, these crystallinity values fall within, or are slightly lower than, the ranges typically reported for hardwood celluloses in the literature [23,24]. Figure 1C compares the DP of celluloses obtained from different hardwood species. Sycamore cellulose exhibits the highest DP value (832.25 ± 15.4), suggesting longer molecular chains and higher average molecular weight, which are generally associated with improved mechanical integrity and structural stability [25]. In contrast, acacia cellulose shows the lowest DP (527 ± 11.8), indicating a higher extent of chain scission, which adversely affects its strength and durability in subsequent processing and application scenarios. The analysis of the infrared crystallinity of the four raw materials showed good agreement with the results of XRD and DP data. Similar FTIR peak positions confirmed that there were no significant differences in the basic chemical structure of cellulose among the samples, while the changes in the intensity of the crystallinity-sensitive peak were consistent with the XRD and DP results.

The TG and DTG curves of the celluloses are shown in Figure 1D and Figure 1E, respectively. All samples display a major mass loss stage between 300 and 400 °C, corresponding to the primary thermal decomposition of cellulose [26]. Acacia cellulose exhibits the fastest degradation rate, with a maximum degradation temperature of 329.9 °C, indicating relatively poor thermal stability. This is followed by eucalyptus (337.5 °C), sycamore (341.3 °C), and poplar (350.8 °C). This behavior is consistent with its lowest DP and lower crystallinity, which suggest a higher proportion of disordered cellulose chains and thermally vulnerable chain segments. The shortening of acacia cellulose chains promotes their thermal decomposition. This is because shorter cellulose chains contain more chain ends and structural defect regions. These regions are more prone to thermal decomposition during pyrolysis. The higher degradation temperatures observed for sycamore and poplar celluloses suggest superior thermal stability, making them more suitable for applications involving elevated processing temperatures [27]. Although acacia cellulose degrades at a lower temperature, it exhibits the highest mass loss rate within the main decomposition region and yields the largest amount of residual char. This behavior is associated with a higher inorganic content or the formation of thermally stable degradation products, which could influence its subsequent valorization pathways. In the temperature range of 400~800 °C, mass loss becomes minimal for all samples, primarily resulting from further bond cleavage and secondary reactions between residual cellulose char and inorganic components. The final residue content, from highest to lowest, is acacia, eucalyptus, sycamore, and poplar. This behavior is likely associated with the inorganic content of cellulose and the formation of non-volatile residues during thermal degradation.

In addition to structural factors, ash content also contributes to the observed thermal behavior. Although sycamore cellulose exhibits relatively high crystallinity and a relatively high degree of polymerization, its thermal stability is slightly lower than that of poplar. This apparent discrepancy suggests that thermal behavior is not governed solely by supramolecular structure, but that it is also influenced by chemical and compositional factors. The ash residue results at 800 °C reveal a higher inorganic content in sycamore (17.32%) compared to poplar (15.79%). Even small differences in inorganic constituents are known to catalyze cellulose pyrolysis by lowering the activation energy for glycosidic bond cleavage and promoting dehydration reactions, thereby shifting thermal degradation to lower temperatures [28,29]. Moreover, the relatively higher accessibility of hydroxyl groups in sycamore cellulose, as indicated by FT-IR analysis, facilitates stronger interactions between cellulose chains and inorganic ions, further enhancing catalytic degradation effects. Therefore, the slightly reduced thermal stability of sycamore compared to poplar can be attributed to the combined influence of inorganic catalysis and hydroxyl accessibility, which offsets the stabilizing effect of higher crystallinity and DP. Although acacia cellulose degrades at a lower temperature, it exhibits the highest mass loss rate within the main decomposition region and yields the largest amount of residual char. This behavior is consistent with its highest ash content (22.42%), suggesting that inorganic components not only catalyze thermal degradation but also promote char formation through secondary reactions, leading to increased residual mass. Therefore, compared to poplar, sycamore has slightly lower thermal stability. This can be attributed to the combined effects of inorganic catalysis and hydroxyl accessibility. This partially offsets the stabilizing effect of its higher degree of polymerization and crystallinity. Overall, the thermal behavior of the celluloses was determined by the combined effects of DP, crystallinity, hydroxyl accessibility, and inorganic composition. Structural factors such as a high degree of polymerization and high crystallinity generally improve thermal stability. Higher inorganic content, on the other hand, promotes catalytic degradation and char formation.

### 3.3. Structural Features and Alkali Sensitivity of Hemicellulose

**Figure 2:**
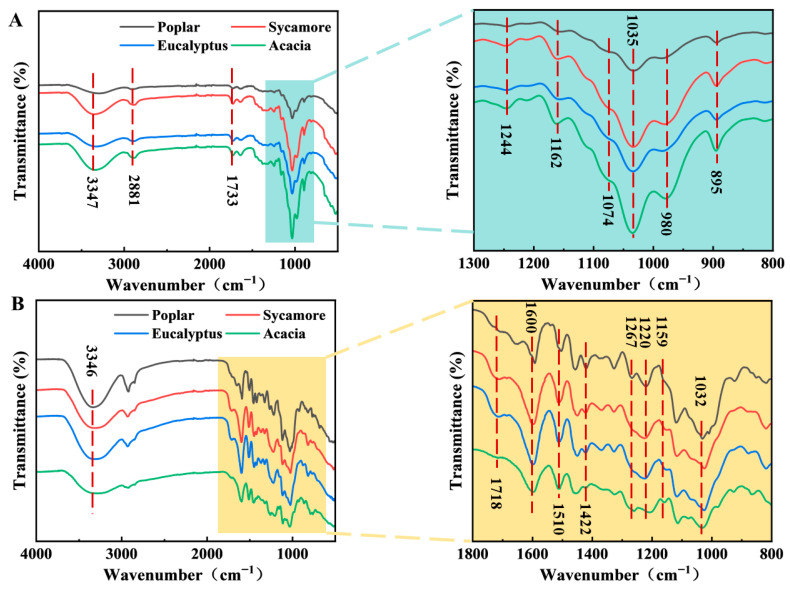
FT-IR spectra of the (**A**) hemicellulose and (**B**) lignin fractions isolated from four hardwood species.

Figure 2 presents the FT-IR spectra of hemicellulose and lignin isolated from hardwood species, revealing distinct characteristic absorption bands associated with hemicellulose structure. These spectral features reflect pronounced interspecies differences in chemical composition and molecular architecture.

The broad absorption band observed at 3400~3200 cm^−1^ is attributed to O-H stretching vibrations, arising from free hydroxyl groups, as well as intra- and intermolecular hydrogen bonding within hemicellulose chains. Notably, this band exhibits higher intensity for sycamore- and acacia-derived hemicelluloses, suggesting a denser hydrogen-bonding network. Such structural features influence moisture adsorption behavior and susceptibility to biodegradation, thereby affecting fiber softening during CMP pretreatment. The absorption peak at 1733 cm^−1^ corresponds to C=O stretching vibrations of acetyl and carboxyl groups in hemicellulose. These functional groups are particularly sensitive to alkaline hydrolysis during CMP processing. Although this peak is present in all samples, its relatively lower intensity in eucalyptus and poplar indicates a reduced acetyl group content, implying enhanced alkali resistance and potentially different softening responses during pretreatment. The absorption peak at 1035 cm^−1^ is characteristic of xylan structures, primarily associated with C-O and C-O-C stretching vibrations within the polysaccharide backbone. The weaker absorption in poplar- and eucalyptus-derived hemicelluloses suggests lower xylan content or structural variations in their hemicellulose fractions [30]. Meanwhile, the absorption peaks at 1162 and 980 cm^−1^ are due to asymmetric C-O-C stretching between α-L-Arap and β-D-Xylp units, representing the presence of arabinosyl side chains in the hemicellulose. The particularly strong absorption observed for acacia-derived hemicellulose indicates a higher arabinoxylan content, which enhances alkali accessibility and facilitates fiber wall softening during pretreatment.

The absorption peak at 895 cm^−1^ represents the C-H deformation vibration of the β-glycosidic bonds between sugar units. The higher intensity of the absorption peak in acacia wood indicates a high concentration of β-1,4-glycosidic bonds in its hemicellulose molecules. This suggests a higher molecular weight and potentially greater structural stability. The absorption peaks at 3347, 2881, 1460, and 1244 cm^−1^ correspond to -OH stretching vibration, symmetrical C-H stretching vibration, C-H bending vibration, and C-O bond stretching vibration, respectively. These peaks are also associated with the basic structural characteristics of hemicellulose, further confirming the differences in the chemical structure and composition of hemicelluloses found in different woods. FT-IR analysis reveals substantial variations in hemicellulose composition and molecular structure among the investigated hardwoods. Acacia exhibits the highest arabinoxylan content and β-1,4-glycosidic linkage density, whereas eucalyptus and poplar contain fewer acetyl groups and lower xylan content. These species-dependent structural differences are expected to influence hemicellulose reactivity under alkaline conditions, thereby playing a critical role in fiber softening behavior, inter-fiber bonding, and the overall performance of alkali-assisted CMP processes.

### 3.4. Functional Group Distribution and Aromatic Architecture of Lignin

Figure 2B displays the FT-IR spectra of lignin isolated from the four hardwood species, revealing distinct characteristic absorption bands associated with lignin chemical structure and functional group distribution. The broad absorption band observed at 3400–3200 cm^−1^ is attributed to O-H stretching vibrations, primarily originating from phenolic and aliphatic hydroxyl groups in lignin [31]. All samples exhibit strong absorption in this region, indicating a relatively high hydroxyl content. Such functional groups play a critical role in lignin chemical reactivity, intermolecular interactions, and potential bonding behavior during biomass processing and utilization. The absorption peak at 1718 cm^−1^ is attributed to the stretching vibration of the C=O bond, representing non-conjugated carboxylic acid, ester, or lactone structures. This peak is more pronounced in sycamore- and eucalyptus-derived lignins, suggesting a higher abundance of carbonyl-containing moieties in these samples. The presence of such groups influences lignin reactivity and thermal behavior under alkaline or thermochemical processing conditions.

Characteristic aromatic skeletal vibrations of lignin are observed at 1600, 1510, and 1421 cm^−1^, corresponding to C=C stretching within the aromatic rings. The band at 1600 cm^−1^ confirms the fundamental aromatic nature of lignin, while the peak at 1510 cm^−1^ is associated with guaiacyl (G) and syringyl (S) units. Eucalyptus lignin exhibits relatively stronger absorption at this position, indicating a higher abundance of G and S structures, which contributes to enhanced aromaticity and thermal stability. The band at 1421 cm^−1^ further supports the presence of aromatic rings in lignin. The absorption peak at 1267 cm^−1^ is attributed to the C-O stretching vibration of the methoxyl groups on the aromatic ring. This represents the characteristic absorption peak of the guaiacyl nucleus methoxyl group and indicates the presence of guaiacyl groups in lignin. The absorption peak at 1220 cm^−1^ is associated with aromatic C-O stretching, particularly characteristic of syringyl units. This peak is relatively stronger in poplar lignin, suggesting a higher syringyl content, which is often correlated with increased lignin reactivity and biodegradability. The absorption peak at 1159 cm^−1^ corresponds to stretching vibrations of C-O-C bonds, reflecting the presence of ether bonds in lignin. The relatively high abundance of ether bonds in acacia lignin suggests that acacia lignin is structurally more compact and stable. This contributes to its improved thermal stability and mechanical properties. The absorption peak at 1032 cm^−1^ is attributed to C-O stretching vibrations, associated with primary and hydroxyl groups, reflecting the hydroxyl content in lignin. The presence of this peak indicates that lignin from all wood samples contains a certain amount of hydroxyl groups, particularly in acacia lignin. This affects its biodegradability and reactivity with other biomass materials.

Infrared analysis reveals significant differences in the composition and structure of hemicellulose and lignin among different wood species. The hemicelluloses of sycamore and acacia woods have stronger hydrogen bond networks, which affects their mechanical properties and biodegradability. Acacia wood hemicellulose has a higher content of arabinoxylan and a larger molecular weight, which contribute to its higher solubility and thermal stability. Eucalyptus and poplar wood hemicelluloses differ in xylan content and carbonyl structure, which affects their applications in industry and the environment. The higher content of carboxylic acid, ester, or lactone groups in the lignins of sycamore and eucalyptus woods contributes to their chemical reactivity. Eucalyptus wood is rich in guaiacyl and syringyl structures, which impart better aromaticity and thermal stability. The more prominent syringyl structure in poplar wood lignin contribute to its biodegradability. The ether bonds and higher hydroxyl content in acacia wood lignin indicate a more compact lignin structure and higher thermal stability. Overall, the structural differences in lignin from different wood species have significant implications for their performance in biomass utilization, environmental applications, and industrial processing.

### 3.5. Molecular Architecture of Hemicellulose, Lignin, and LCCs

**Figure 3:**
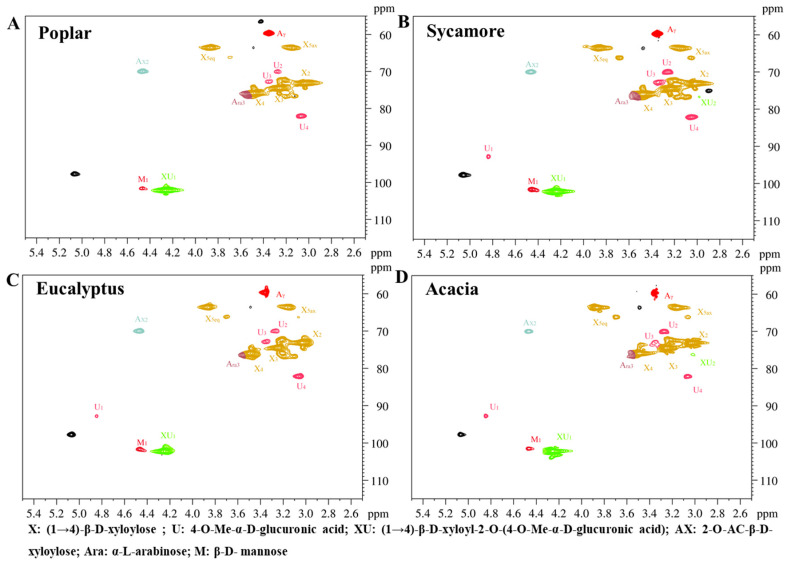
2D-NMR spectroscopic analysis and structural representation of hemicellulose components. (**A**) Poplar, (**B**) sycamore, (**C**) eucalyptus, and (**D**) acacia.

Hemicellulose, lignin, and LCCs from the four raw materials were extracted and analyzed by two-dimensional nuclear magnetic resonance (NMR). The NMR spectra of the hemicellulose (Figure 3A–D) indicate that hemicelluloses from poplar, sycamore, eucalyptus, and acacia share a common backbone composed predominantly of β-(1→4)-linked xylopyranosyl units, accompanied by characteristic side-chain signals assigned to acetyl groups and 4-O-methyl-α-D-glucuronic acid substituents. These features confirm that the hemicelluloses exhibit typical glucuronoxylan structures with relatively high degrees of polymerization and structural stability. Despite the similarity of the main-chain architecture, pronounced species-dependent differences were observed in the degree of side-chain substitution. Relatively stronger acetyl signals were detected in poplar and acacia hemicelluloses (Figure 3A,D), indicating higher levels of acetylation, which are known to enhance water solubility and chemical reactivity [32]. In contrast, weaker acetyl signals were observed for sycamore and eucalyptus (Figure 3B,C), suggesting lower acetylation degrees and, consequently, reduced solubility of their hemicelluloses. Signals corresponding to 4-O-methyl-α-D-glucuronic acid side chains were more pronounced in poplar and eucalyptus (Figure 3A,C), implying a higher abundance of glucuronic acid substitutions that can promote interactions and cross-linking with lignin. Conversely, the weaker glucuronic acid signals observed in acacia and sycamore hemicelluloses (Figure 3B,D) indicate a lower degree of such substitutions, which limits the formation of LCCs. These variations in acetylation and glucuronation patterns are expected to influence the chemical reactivity, interpolymer interactions, and pulping behavior of hemicelluloses during alkali-assisted CMP processing.

**Figure 4:**
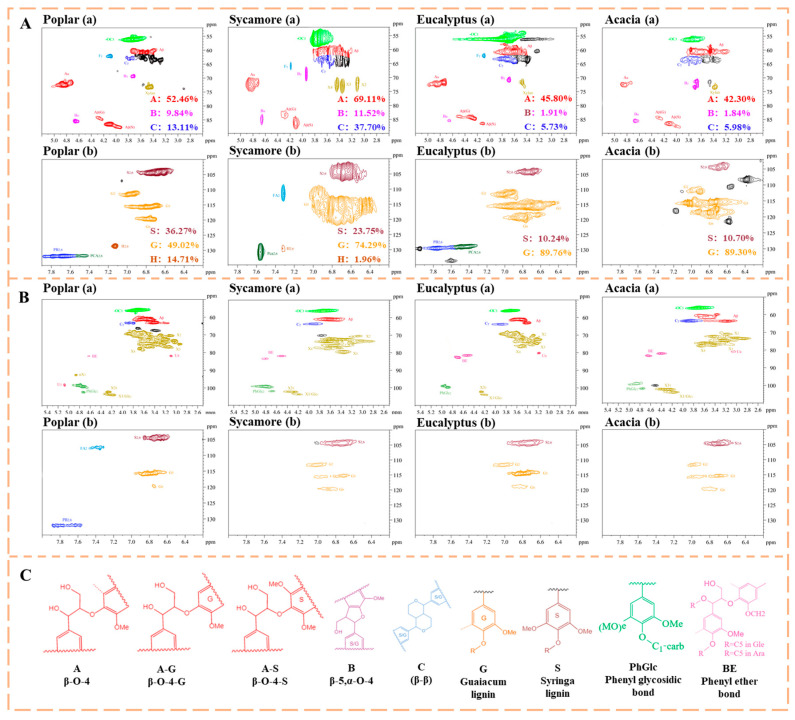
2D-NMR spectroscopic analysis and structural representation of hardwood components. (**A**) Lignin, (**B**) LCC, and (**C**) a schematic diagram of representative molecular structures and key inter-unit linkages.

The 2D NMR spectra of lignin isolated from the four hardwood species are presented in Figure 4A. The spectra are dominated by characteristic signals associated with aromatic rings, methoxy groups, and inter-unit ether linkages, which are typical structural features of hardwood lignin [33]. Signals in the aromatic region (δ 6.0~7.0 ppm) are mainly assigned to C-H correlations of guaiacyl (G) and syringyl (S) units, whereas signals in the δ 3.5~4.0 ppm region correspond primarily to methoxyl groups. Poplar lignin exhibits strong aromatic signals indicating a high abundance of G and S units, which contributes to its pronounced aromaticity and potentially enhanced thermal stability [34]. In contrast, sycamore lignin displays relatively weaker aromatic signals, suggesting a lower content of G and S structures and a comparatively simpler aromatic framework. Eucalyptus lignin shows aromatic signal intensities comparable to those of poplar, implying a similarly high content of G and S units and associated aromatic stability [35]. Acacia lignin also exhibits intense aromatic signals, indicating a high concentration of aromatic structural units. Poplar and eucalyptus lignins show relatively strong methoxyl signals, suggesting a higher degree of methoxyl substitution, which is generally associated with increased chemical reactivity during alkaline processing. In contrast, sycamore lignin presents weaker methoxyl signals, indicating fewer methoxyl groups and potentially reduced reactivity. Acacia lignin exhibits pronounced ether-related correlations, reflecting a higher abundance of ether linkages, which contribute to its enhanced chemical and structural stability.

The LCC fractions display mixed spectral features characteristic of both lignin and hemicellulose, as shown in Figure 4B. Strong aromatic signals coupled with carbohydrate-related correlations are observed in the LCC spectra of poplar, eucalyptus, and acacia, indicating extensive cross-linking between lignin aromatic units and hemicellulose sugar moieties [36]. In contrast, the LCC spectrum of sycamore exhibits relatively weak aromatic signals, suggesting a lower degree of lignin-carbohydrate cross-linking and a looser association between lignin and hemicellulose.

Overall, poplar and eucalyptus are characterized by higher hemicellulose contents, lignins rich in aromatic and methoxyl structures, and stronger LCC cross-linking, indicating densely packed and chemically interactive lignocellulosic networks. Sycamore lignin exhibits lower aromaticity, fewer methoxyl substitutions, and weak LCC signals, reflecting a less complex and more weakly bound lignin-hemicellulose structure. Acacia lignin, while highly aromatic, is distinguished by abundant ether linkages and strong LCC correlations, suggesting superior structural stability.

### 3.6. Physical Accessibility and Microstructural Constraints of Hardwood Fibers

**Figure 5:**
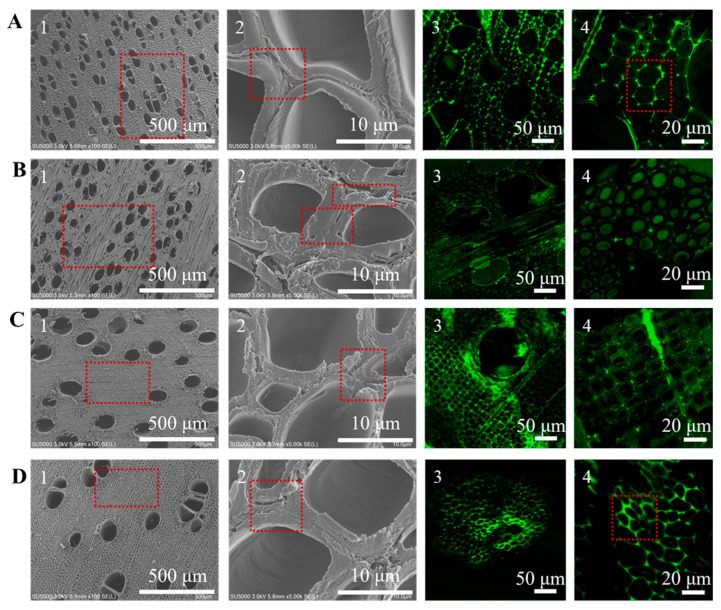
Microscopy and confocal laser scanning microscopy of the four hardwood species. (**A**) Poplar, (**B**) sycamore, (**C**) eucalyptus, and (**D**) acacia.

The physical properties and microstructural characteristics of the four hardwood species were systematically analyzed, and the results are summarized in Table 2 and Figure 5. Pronounced species-dependent differences in density, porosity, pore size distribution, and fiber cell wall structure were observed, which are expected to play a critical role in chemical penetration, fiber softening, and mechanical refining behavior during CMP processing.

As shown in Table 2, poplar exhibits the lowest density (342.53 ± 9.85 kg/m^3^) and the highest porosity (66.86 ± 1.25%) among the four species, together with a relatively large average pore diameter (898.60 ± 16.56 nm). These characteristics facilitate rapid water uptake and chemical penetration through capillary action and vessel networks, resulting in a high water absorption rate (171.81 ± 5.51%). In addition, poplar fibers possess thin cell walls (1.47 ± 0.25 μm) and a relatively large fiber lumen diameter (18.04 ± 1.04 μm), leading to a loose fiber network with reduced resistance to deformation. Such a microstructural configuration promotes efficient fiber softening and lignin dissolution, thereby facilitating fiber separation and reducing the energy demand during mechanical refining.

Sycamore exhibits a higher density (398.14 ± 10.42 kg/m^3^) and lower porosity (60.20 ± 1.06%) compared with poplar, indicating a more compact fiber structure. Its smaller average pore diameter (73.76 ± 1.02 nm) and thicker cell walls (2.03 ± 0.32 μm) hinder chemical penetration into the fiber matrix, despite a relatively high water absorption rate (194.06 ± 8.25%) driven by the strong hydrophilicity of cellulose and hemicellulose. The smaller fiber lumen diameter (13.29 ± 1.05 μm) results in a higher number of fiber cells per unit area, further increasing structural compactness. These features limit chemical softening during CMP pretreatment, resulting in higher refining energy consumption and an increased risk of fiber damage [37].

Eucalyptus displays the highest density (432.77 ± 15.23 kg/m^3^) and the lowest porosity (52.96 ± 1.01%) among the four hardwoods, reflecting a highly compact wood structure. This intrinsic compactness makes eucalyptus particularly resistant to chemical penetration during alkali-assisted CMP [38]. The low porosity and moderate water absorption rate (155.52 ± 6.56%) indicate slow liquid uptake, necessitating stronger chemical action to disrupt the cell wall matrix and facilitate lignin removal. Although the cell wall thickness of eucalyptus fibers (1.45 ± 0.18 μm) is comparable to that of poplar, the smaller fiber lumen diameter results in a denser fiber assembly, leading to localized fiber softening near vessel regions while leaving a large proportion of fibers insufficiently treated. Consequently, eucalyptus requires higher mechanical energy input and is more prone to fiber damage during refining.

Acacia exhibits an intermediate density (356.79 ± 10.04 kg/m^3^), lower than that of eucalyptus and sycamore but higher than that of poplar. Its porosity (65.50 ± 1.32%) is comparable to poplar; however, the significantly smaller average pore diameter (69.35 ± 1.45 nm) indicates a more compact pore structure. The relatively low water absorption rate (159.68 ± 5.25%) and moderate fiber lumen diameter (13.01 ± 0.99 μm) suggest a dense fiber arrangement with limited internal accessibility. As a result, chemical penetration during CMP pretreatment is restricted, leading to incomplete fiber softening and increased mechanical energy demand during refining, similar to the behavior observed for eucalyptus.

The microstructural features of the four hardwood species are further illustrated by microscopy and confocal laser scanning microscopy images in Figure 5. All untreated raw materials exhibit thick cell walls and intercellular regions filled with non-cellulosic materials, primarily lignin, hemicellulose, and pectin, with lignin being predominantly concentrated in the middle lamella and cell corners. Poplar (Figure 5A) shows a loose, ring-porous structure with large vessels and intercellular spaces, which favors rapid chemical penetration and effective fiber separation. In contrast, sycamore (Figure 5B) presents a denser microstructure with thicker cell walls and reduced intercellular spaces, limiting chemical accessibility. Eucalyptus (Figure 5C) exhibits a highly compact cell arrangement with low porosity, further restricting liquid transport and chemical diffusion. Acacia (Figure 5D) displays microstructural features intermediate between poplar and eucalyptus, with moderate fiber length and porosity but small pore sizes and densely packed intercellular regions, which impede chemical penetration and fiber dissociation.

Fluorescence imaging reveals strong signals in the middle lamella and cell corners for all four species, confirming that lignin is predominantly localized in these regions [39]. This highly lignified middle lamella imparts mechanical strength and rigidity to the wood but also constitutes the most resistant barrier during pulping, emphasizing the importance of effective chemical pretreatment strategies to disrupt lignin-rich interfaces and facilitate fiber separation in CMP processing.

### 3.7. Alkali-Assisted Fiber Softening and Microstructural Evolution

**Figure 6:**
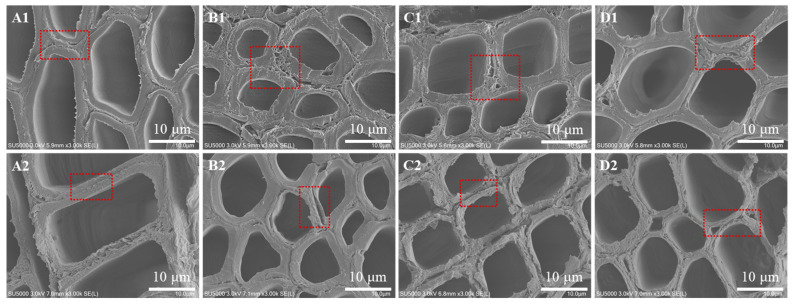
Comparative microstructural evolution of hardwood fibers before and after alkali steaming treatment. (**A1**,**A2**) Poplar, (**B2**,**B2**) sycamore, (**C1**,**C2**) eucalyptus, and (**D1**,**D2**) acacia.

The microstructural evolution of the four hardwood species before and after alkali steaming treatment is illustrated in Figure 6. Prior to steaming, all untreated woods exhibit compact and well-organized cellular architectures, characterized by thick fiber cell walls and intercellular regions densely filled with highly lignified materials, particularly in the middle lamella and cell corners. These lignin-rich domains impart high mechanical strength and rigidity to the wood but also constitute a major barrier to chemical penetration and fiber softening during pulping, thereby limiting processability.

Following alkali steaming treatment, pronounced structural modifications were observed in all four species. The fiber cell walls became significantly thinner, accompanied by partial disruption of the intercellular layers. This reduction in cell wall thickness indicates effective removal or redistribution of lignin and associated matrix components under alkaline conditions, leading to decreased structural compactness and enhanced fiber flexibility. As a result, fiber softening is substantially improved, creating more favorable conditions for subsequent mechanical refining. Among the four species, sycamore exhibits the most pronounced response to alkali steaming. As shown in Figure 6(B1,B2), the fiber cell wall thickness is reduced to approximately 40~50% of that of the untreated material, reflecting extensive degradation of lignified structures. This pronounced susceptibility is attributed to the initially thicker cell walls and higher lignin concentration in sycamore fibers, which provide more reactive sites for alkaline attack. The effective weakening of these rigid structures facilitates subsequent fiber separation and softening, partially compensating for the inherent resistance of sycamore wood during CMP processing [40].

### 3.8. Species-Dependent Response to Alkali Charge in CMP

**Figure 7:**
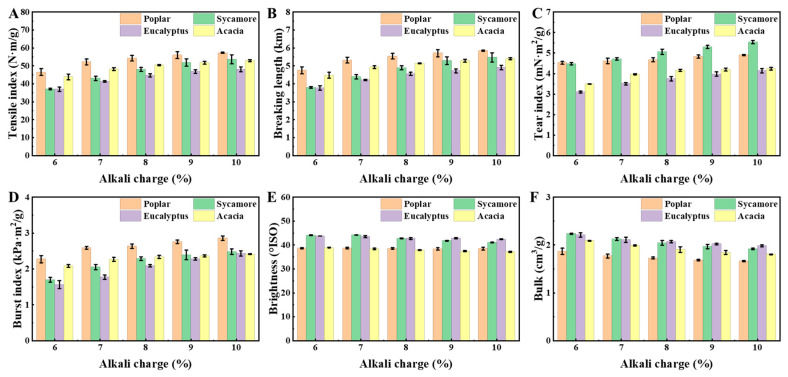
Hardwood chemical-mechanical pulp paper strength. (**A**) Tensile index, (**B**) breaking length, (**C**) tear index, (**D**) burst index, (**E**) brightness, and (**F**) bulk.

To evaluate the influence of alkali charge on hardwood CMP performance, pulp preparation was systematically investigated under different alkali conditions. The papermaking properties of poplar, sycamore, eucalyptus, and acacia pulps, refined to a beating degree of 40 ± 2 °SR, were evaluated, with the results summarized in Figure 7. In addition to paper strength properties, the influence of alkali charge on pulp fractionation behavior was further evaluated in terms of accepted pulp yield and coarse pulp yield, as summarized in Table 3. With increasing NaOH charge, the accepted pulp yield increases consistently, while the coarse pulp yield decreases markedly for all four hardwood species. This trend indicates that higher alkali charge enhances fiber separation efficiency during refining, promoting the conversion of fiber bundles into individual fibers. The total yield shows a slight decreasing tendency with increasing alkali charge, which can be attributed to the dissolution of hemicellulose and partial degradation of lignin under alkaline conditions. Notably, pronounced species-dependent differences are observed. At low alkali charge (6%), eucalyptus exhibits the highest coarse pulp yield (32.74 ± 1.04%), indicating more resistant fiber separation, whereas poplar shows relatively lower coarse pulp content (25.33 ± 0.59%), suggesting better initial accessibility and fiber liberation. This difference is consistent with the denser and more compact cell wall structure of eucalyptus compared to the more porous and permeable structure of poplar. With increasing alkali charge, these differences gradually diminish, indicating that higher alkali input can partially overcome structural constraints and improve fiber separation efficiency across all species.

As shown in Figure 7A–D, increasing the NaOH charge from 6% to 10% leads to a pronounced enhancement in tensile index, breaking length, tear index, and burst index for all four hardwood pulps. This trend demonstrates that appropriately intensified alkali-assisted CMP can effectively improve fiber bonding and enhance paper strength. Meanwhile, bulk decreases with increasing alkali charge (Figure 7F), although the magnitude of this reduction varies substantially among species. Overall, moderate alkali addition improves fiber bonding and paper strength, consistent with previous reports [41].

The observed improvement in mechanical properties can be attributed to the enhanced fiber softening induced by higher alkali charges. Alkaline treatment promotes partial dissolution of hemicellulose and lignin, loosening the fiber wall structure and facilitating fiber separation during refining. This reduces excessive mechanical damage, preserves fiber integrity, and, ultimately, enhances paper strength [42]. However, excessive alkali charge leads to over-dissolution of hemicellulose, which reduces bonding potential and adversely affect the strength performance of the resulting paper [43].

Notably, the response to alkali charge exhibits clear species-dependent behavior. For poplar and acacia, further increases in NaOH charge beyond 8% result in a diminished rate of strength improvement. In contrast, sycamore and eucalyptus maintain a positive response until the NaOH charge reaches approximately 9%, after which strength gains level off. This distinction highlights the importance of species-specific optimization in alkali-assisted CMP of different hardwood species. The higher alkali demand of sycamore is attributed to its relatively rigid fiber structure, which limits alkali-induced softening. Similarly, eucalyptus requires higher alkali charge due to its dense and compact fiber architecture, which restricts chemical penetration. In contrast, poplar exhibits a more porous structure that facilitates alkali diffusion, allowing effective pulping at comparatively lower alkali levels. Among the four species, poplar CMP consistently exhibits superior paper strength, confirming that hardwoods with favorable intrinsic anatomical features can retain high pulping potential under mild alkaline processing conditions. The loose microstructure, thin cell walls, and abundant pores in poplar facilitate rapid and homogeneous alkali penetration, resulting in high alkali uptake and well-preserved fiber integrity. Consequently, poplar CMP fibers remain relatively long and robust, contributing to enhanced paper strength performance.

### 3.9. Fiber Quality Evolution and Fibrillation Behavior Under Mechanical Refining

Further fiber quality analysis (Table 4) reveals that increasing NaOH charge promotes an increase in average fiber length and aspect ratio, accompanied by a reduction in fines content. This trend is particularly important for hardwood CMP, where preserving fiber length is essential for achieving favorable paper strength. Alkali-induced selective swelling and softening of the fiber wall reduce tearing, shearing, and secondary fiber shortening during mechanical refining. As a result, mechanical friction and damage are alleviated, enabling higher fiber length retention and suppressing fines generation. Consistent with paper strength results, the positive effect on fiber length diminishes beyond 7% NaOH for poplar and acacia and beyond 9% for sycamore and eucalyptus, further confirming the existence of optimal alkali thresholds for different hardwood species.

**Figure 8:**
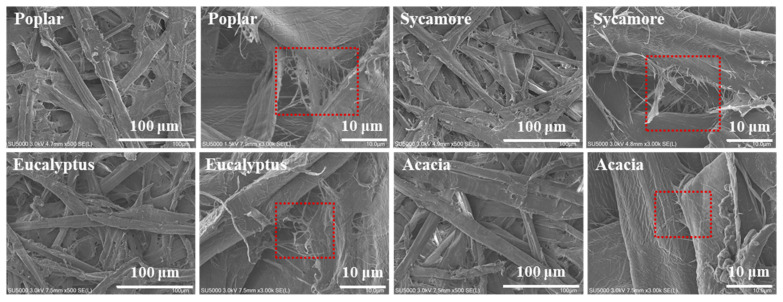
Differential effects of 9% NaOH concentration on the microstructure of fibers from various hardwood species.

To verify the above hypothesis, SEM examination was employed to examine the surface morphology of the prepared papers, and the microstructural differences under varying alkali charges were systematically compared. Representative SEM images are presented in Figures S1–S3 and Figure 8. As shown in Figure S1, when the NaOH charge was 6%, the degree of fiber fibrillation was generally low for all four hardwood pulps, with this effect being particularly pronounced in sycamore and eucalyptus. The fiber surfaces of sycamore and eucalyptus appeared relatively smooth, and the dominant features were surface wrinkling and localized damage rather than well-developed fibrillation. This observation indicates that at low alkali charge, fiber softening was insufficient, as lignin and hemicellulose were not adequately dissolved or plasticized. Consequently, the inter-fiber bonding remained strong, hindering effective fiber separation. In sycamore and eucalyptus, where lignin and other matrix components are more densely interwoven, the compact fiber architecture further limited alkali penetration, resulting in incomplete fiber splitting and dissociation.

With increasing NaOH charge, a pronounced enhancement in fiber fibrillation and brooming was observed. When the NaOH charge reached 8% or higher, evident surface splitting, peeling, and cracking appeared on the fiber surface. These features indicate effective fiber wall softening and a reduction in inter-fiber bonding strength, facilitating the dissociation of fibers into more individualized elements. This morphological evolution reflects the progressive dissolution and softening of lignin and hemicellulose induced by alkali treatment, which substantially improves fiber flexibility and separability.

At a NaOH charge of 8% (Figure S3), poplar and acacia fibers already exhibited extensive fibrillation and surface roughening, accompanied by partial fiber fragmentation. In contrast, sycamore and eucalyptus fibers still showed limited fibrillation and occasional structural damage, suggesting that the alkali intensity remained insufficient for these denser hardwood species. When the NaOH charge increased to 9% (Figure 8), sycamore and eucalyptus fibers became more uniformly softened while largely maintaining structural integrity, and the degree of fibrillation was significantly enhanced. This indicates that a NaOH charge of approximately 9% is more suitable for achieving effective fiber activation in sycamore and eucalyptus, consistent with the trends observed in the corresponding paper strength results. Comparison of SEM images obtained at different alkali charges clearly reveals the progressive development of wrinkling, flaking, and splitting structures on fiber surfaces. The emergence of these surface features increases surface roughness and effective contact area between fibers, thereby promoting inter-fiber bonding and enhancing paper strength. These microstructural observations confirm that the improvement in paper mechanical performance is primarily driven by alkali-induced fiber fibrillation and surface roughening, which are critical for enhancing inter-fiber bonding capacity in hardwood chemical-mechanical pulps.

### 3.10. Crystalline Structure Evolution of CMP Fibers

**Figure 9:**
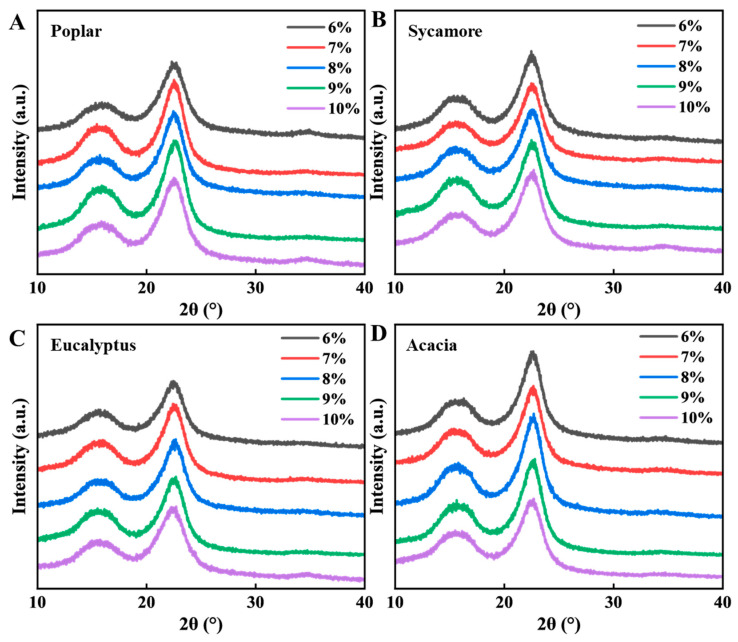
XRD pattern of hardwood chemical-mechanical pulp. (**A**) Poplar, (**B**) sycamore, (**C**) eucalyptus, and (**D**) acacia.

XRD analysis was performed to investigate the crystalline structure evolution of the prepared hardwood chemical-mechanical pulps. The corresponding diffraction patterns are presented in Figure 9. At low alkali charges, the alkali-assisted softening of wood fibers was insufficient, and mechanical forces dominated the pulping process. Under these conditions, fibers were forcibly dissociated and subjected to intensive shearing during mechanical refining, resulting in severe fiber damage. This mechanical disruption partially destroyed the cellulose crystalline regions, leading to a reduction in fiber crystallinity, as evidenced by the weakened diffraction intensity in Figure 9. This result is consistent with the fiber quality and SEM observations discussed above. At insufficient NaOH charge, the fiber wall was not adequately swollen or plasticized, and fiber separation mainly depended on mechanical force. The limited development of surface fibrillation indicated insufficient fiber activation and weak inter-fiber bonding potential. Therefore, the lower crystallinity observed under low alkali loads may be related to excessive mechanical damage to cellulose microfibrils. The correspondingly poor fiber quality further limits the formation of a robust paper network.

With increasing alkali charge, the softening and swelling effect of the alkali solution on the wood fibers was significantly enhanced. Improved fiber plasticization reduced resistance to mechanical action during pulping, thereby alleviating excessive shear and compressive damage to the cellulose microfibrils. As a result, the integrity of the crystalline regions was better preserved, and the crystallinity of the resulting pulp fibers increased, as reflected by the more pronounced crystalline diffraction peaks. More importantly, this crystalline structure evolution is closely related to the improvement in fiber quality. Within the appropriate alkali charge range, alkali treatment not only preserves the cellulose crystalline regions but also promotes fiber wall swelling, surface fibrillation, and brooming. These structural changes increase fiber flexibility, surface roughness, and effective contact area between fibers, thereby enhancing inter-fiber bonding. At the same time, the preservation of fiber length and the reduction in fines content contribute to the formation of a more continuous and mechanically stable fiber network. Therefore, the improvement in paper strength can be attributed to the synergistic effect of crystalline structure preservation and fiber morphological optimization.

However, when the alkali charge was further increased beyond the optimal range, the beneficial softening effect was gradually offset by excessive chemical degradation. Under these conditions, partial dissolution of cellulose and over-removal of amorphous components occurred, disrupting the ordered arrangement of cellulose chains. Consequently, the crystallinity of the pulp decreased again at high alkali charges. Overall, the non-monotonic changes in crystallinity correlated well with the trends in fiber quality and paper strength. Appropriate amounts of sodium hydroxide effectively balanced the fiber softening, fiber length retention, surface fibrillation, and cellulose crystal structure preservation induced by alkali treatment. The combined effect of these structural advantages promoted inter-fiber bonding, thereby improving the mechanical properties of the paper. Conversely, insufficient alkali treatment led to fiber dissociation, primarily causing mechanical damage. Excessive alkali treatment, on the other hand, resulted in chemical degradation and fiber weakening. Therefore, the XRD results further confirm the existence of species-dependent optimal alkali charge windows for hardwood CMP preparation.

## 4. Conclusions

This study systematically investigated the structure-property relationships associated with the alkali-assisted CMP behavior of four representative hardwood species by integrating chemical composition, microstructural characteristics, and pulping response. Distinct species-dependent differences in lignocellulosic architecture, including cellulose supramolecular organization, LCC features, cell wall compactness, and pore structure, were shown to be closely associated with variations in alkali penetration, fiber softening efficiency, and resistance to mechanical refining. These intrinsic structural attributes directly determined the balance between fiber preservation and fibrillation development under mild alkaline CMP conditions, thereby controlling inter-fiber bonding potential and paper strength performance. Importantly, the results demonstrate that appropriate adjustment of alkali charge, in accordance with species-specific chemical and physical properties, can avoid excessive polysaccharide degradation while promoting effective fiber separation. Hardwoods with lower density, higher porosity, and thinner fiber cell walls, exemplified by poplar, exhibited enhanced alkali accessibility and more effective fiber preservation during CMP, resulting in superior fibrillation development and paper strength properties under relatively mild alkaline conditions. In contrast, denser and more compact structures, as observed in sycamore and eucalyptus, required higher alkali charges to achieve sufficient fiber softening and separation, while excessive alkaline treatment led to increased polysaccharide dissolution and diminished strength gains. Acacia displayed intermediate behavior, governed by its compact pore structure and relatively stable lignin architecture. Overall, this work provides an integrated qualitative understanding of the relationships between wood structure, alkali response, and CMP performance, and identifies key structural descriptors associated with species-dependent behavior. These findings offer useful guidance for tailoring alkali-assisted CMP strategies to different hardwood species, contributing to more efficient and sustainable utilization of lignocellulosic biomass. Future work incorporating multivariate or quantitative modeling is needed to further disentangle the relative contributions of these factors.

## Figures and Tables

**Table 1:** Chemical component content of hardwood (%).

Samples	Cellulose	Hemicellulose	Acid Insoluble Lignin	Alcohol Benzene Extractive	Ash
Poplar	43.57 ± 1.23	26.67 ± 1.52	22.18 ± 1.61	2.03 ± 0.06	0.42 ± 0.01
Sycamore	46.67 ± 1.04	21.02 ± 1.41	22.25 ± 1.52	5.01 ± 0.05	0.47 ± 0.03
Eucalyptus	45.96 ± 0.85	23.82 ± 1.62	26.22 ± 1.32	1.66 ± 0.08	0.39 ± 0.02
Acacia	44.95 ± 1.45	22.48 ± 1.25	27.69 ± 1.41	4.96 ± 0.04	0.51 ± 0.03

**Table 2:** Wood’s physical properties.

Properties	Poplar	Sycamore	Eucalyptus	Acacia
Density (kg/m^3^)	342.53 ± 9.85	398.14 ± 10.42	432.77 ± 15.23	356.79 ± 10.04
Porosity (%)	66.86 ± 1.25	60.20 ± 1.06	52.96 ± 1.01	65.50 ± 1.32
Average pore size (nm)	898.60 ± 16.56	73.76 ± 1.02	116.28 ± 1.18	69.35 ± 1.45
Median pore size V (nm)	17,903.07 ± 58.52	175.85 ± 1.15	331.90 ± 3.85	77.77 ± 0.99
Total pore volume (mL/g)	1.78 ± 0.14	1.18 ± 0.08	0.83 ± 0.11	1.44 ± 0.15
Water absorption (%)	171.81 ± 5.51	194.06 ± 8.25	155.52 ± 6.56	159.68 ± 5.52
Effective alkali (%)	13.33 ± 0.15	15.24 ± 0.21	22.86 ± 0.18	18.85 ± 0.16
Fiber length (mm)	0.79 ± 0.03	1.08 ± 0.04	0.69 ± 0.03	0.74 ± 0.02
Fiber width (μm)	22.6 ± 0.12	24.9 ± 0.09	19.7 ± 0.12	19.6 ± 0.08
Fiber hardness (MPa)	232.51 ± 12.21	158.30 ± 9.52	166.11 ± 10.85	148.27 ± 13.24
Cell wall thickness (μm)	1.47 ± 0.25	2.03 ± 0.32	1.45 ± 0.18	0.61 ± 0.15
Lumen diameter (μm)	18.04 ± 1.04	10.27 ± 0.98	14.58 ± 0.87	13.01 ± 0.99
Wall-to-cavity ratio	0.21 ± 0.02	0.29 ± 0.02	0.14 ± 0.01	0.31 ± 0.02
Fiber cell diameter (μm)	21.78 ± 1.15	13.29 ± 1.05	16.56 ± 0.95	17.04 ± 1.08

**Table 3:** The accepted pulp and coarse pulp yield of hardwood chemical-mechanical pulps as a function of NaOH charge.

Alkali Charge (%)	Poplar (%)		Sycamore (%)		Eucalyptus (%)		Acacia (%)	
	Accepted Pulp	Coarse Pulp	Accepted Pulp	Coarse Pulp	Accepted Pulp	Coarse Pulp	Accepted Pulp	Coarse Pulp
6	57.11 ± 1.04%	25.33 ± 0.59%	47.43 ± 0.98%	27.76 ± 0.67%	39.18 ± 1.05%	32.74 ± 1.04%	49.63 ± 1.31%	25.13 ± 0.94%
7	65.94 ± 1.16%	18.73 ± 0.77%	53.18 ± 0.97%	25.09 ± 0.76%	45.03 ± 0.94%	29.72 ± 0.97%	62.55 ± 1.18%	17.84 ± 0.89%
8	69.52 ± 0.89%	14.18 ± 0.69%	55.66 ± 1.21%	23.69 ± 0.82%	54.24 ± 0.99%	24.63 ± 0.75%	64.63 ± 0.98%	13.82 ± 0.75%
9	70.27 ± 0.96%	11.93 ± 0.81%	59.09 ± 1.11%	17.06 ± 0.74%	60.20 ± 1.02%	17.87 ± 0.82%	66.36 ± 1.14%	11.73 ± 0.92%
10	71.06 ± 0.88%	10.14 ± 0.65%	61.36 ± 0.99%	12.65 ± 0.68%	62.22 ± 1.02%	15.20 ± 0.84%	68.48 ± 1.04%	7.82 ± 0.79%

**Table 4:** Fiber dimensions of hardwood chemical-mechanical pulps as a function of NaOH charge.

Sample	Alkali Charge (%)	Length (mm)	Width (μm)	Aspect Ratio	Fines Ratio (%)
Poplar	6	0.55 ± 0.02	27.6 ± 0.04	19.86 ± 0.03	9.67 ± 0.18
	7	0.58 ± 0.04	27.2 ± 0.02	21.08 ± 0.02	8.15 ± 0.11
	8	0.59 ± 0.03	27.8 ± 0.06	21.18 ± 0.03	7.96 ± 0.15
	9	0.57 ± 0.02	26.8 ± 0.05	21.19 ± 0.03	7.68 ± 0.08
	10	0.56 ± 0.02	26.4 ± 0.02	21.24 ± 0.02	7.46 ± 0.13
Sycamore	6	0.59 ± 0.03	27.9 ± 0.03	21.00 ± 0.02	10.4 ± 0.16
	7	0.62 ± 0.02	27.7 ± 0.04	22.38 ± 0.02	9.43 ± 0.09
	8	0.65 ± 0.04	27.8 ± 0.02	23.31 ± 0.03	9.08 ± 0.11
	9	0.67 ± 0.05	27.8 ± 0.05	24.35 ± 0.02	8.83 ± 0.15
	10	0.68 ± 0.03	27.6 ± 0.03	24.53 ± 0.01	8.68 ± 0.012
Eucalyptus	6	0.47 ± 0.02	23.4 ± 0.04	20.01 ± 0.02	13.35 ± 0.07
	7	0.48 ± 0.05	22.3 ± 0.02	21.30 ± 0.03	11.05 ± 0.09
	8	0.50 ± 0.02	22.6 ± 0.05	22.08 ± 0.03	10.54 ± 0.12
	9	0.52 ± 0.03	23.2 ± 0.01	22.37 ± 0.01	10.02 ± 0.25
	10	0.53 ± 0.02	22.8 ± 0.04	23.38 ± 0.02	9.84 ± 0.09
Acacia	6	0.49 ± 0.05	22.7 ± 0.01	21.45 ± 0.01	9.14 ± 0.08
	7	0.51 ± 0.02	22.2 ± 0.07	22.84 ± 0.03	8.98 ± 0.02
	8	0.51 ± 0.04	22.5 ± 0.04	22.76 ± 0.02	8.48 ± 0.07
	9	0.52 ± 0.04	22.3 ± 0.05	22.96 ± 0.03	8.09 ± 0.14
	10	0.52 ± 0.03	22.1 ± 0.03	23.58 ± 0.01	7.76 ± 0.12

## Data Availability

The data are not publicly available due to privacy or ethical restrictions, data will be made available on request.

## Supplementary Materials

The following supporting information can be downloaded at: https://www.mdpi.com/article/10.3390/polym18101195/s1, Figure S1: Differential effects of 6% NaOH concentration on the microstructure of fibers from various hardwood species; Figure S2: Differential effects of 7% NaOH concentration on the microstructure of fibers from various hardwood species; Figure S3: Differential effects of 8% NaOH concentration on the microstructure of fibers from various hardwood species.
