# Supplementary material for: Structure-Property Relationships Governing Species Dependent Response in Alkali-Assisted Chemical-Mechanical Pulping of Hardwoods
Source: Polymers (Basel). 2026 May 13;18(10):1195. doi: 10.3390/polym18101195 (PMC13210677; doi:10.3390/polym18101195)
Supplement: Supplementary file 1 [file polymers-18-01195-s001.zip › polymers-4242922-supplementary.pdf]

Structure-Property Relationships Governing Species Dependent Response in Alkali-Assisted Chemical-Mechanical Pulping of Hardwoods

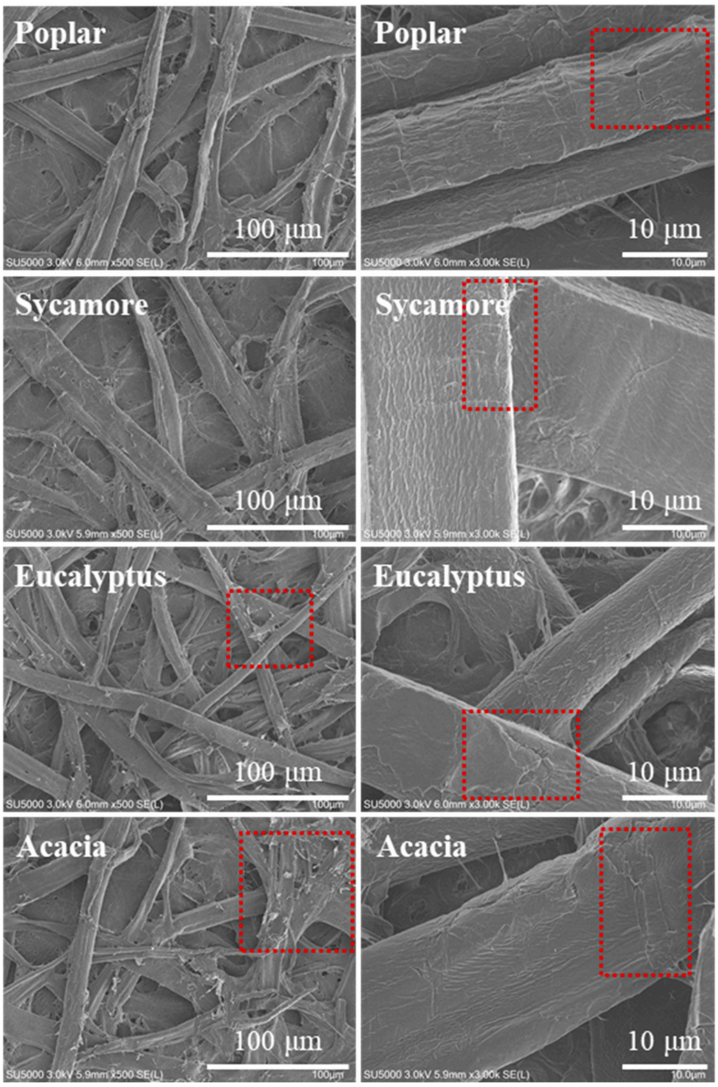

Figure S1. Differential effects of 6% NaOH concentration on the microstructure of fibers from various hardwood species

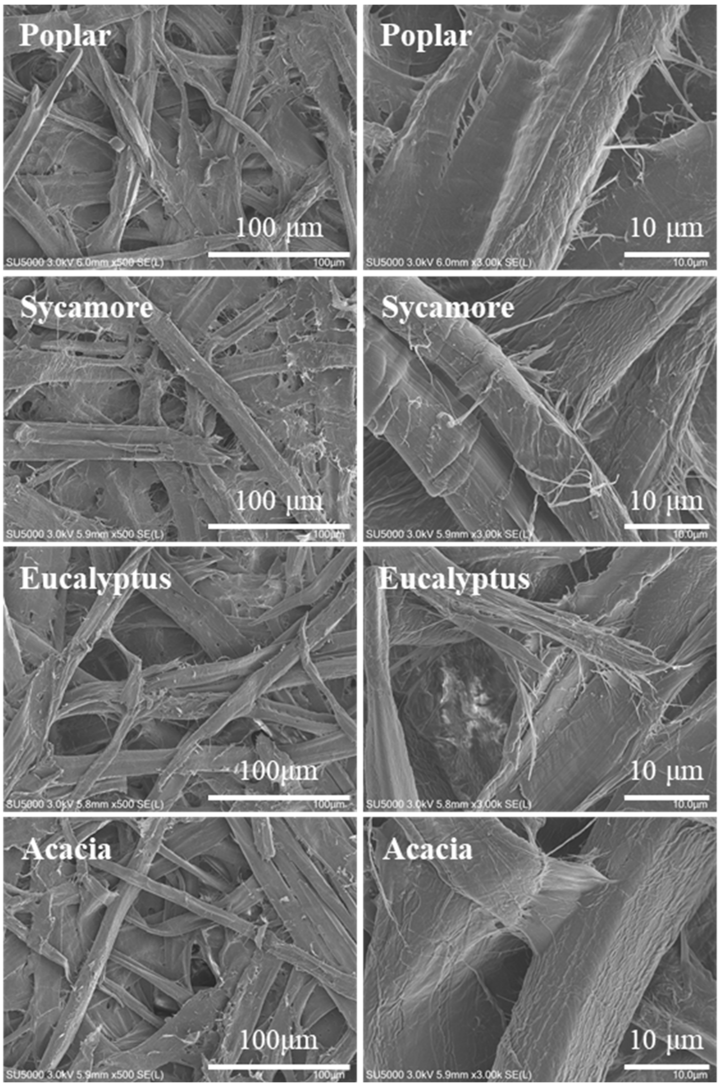

Figure S2. Differential effects of 7% NaOH concentration on the microstructure of fibers from various hardwood species

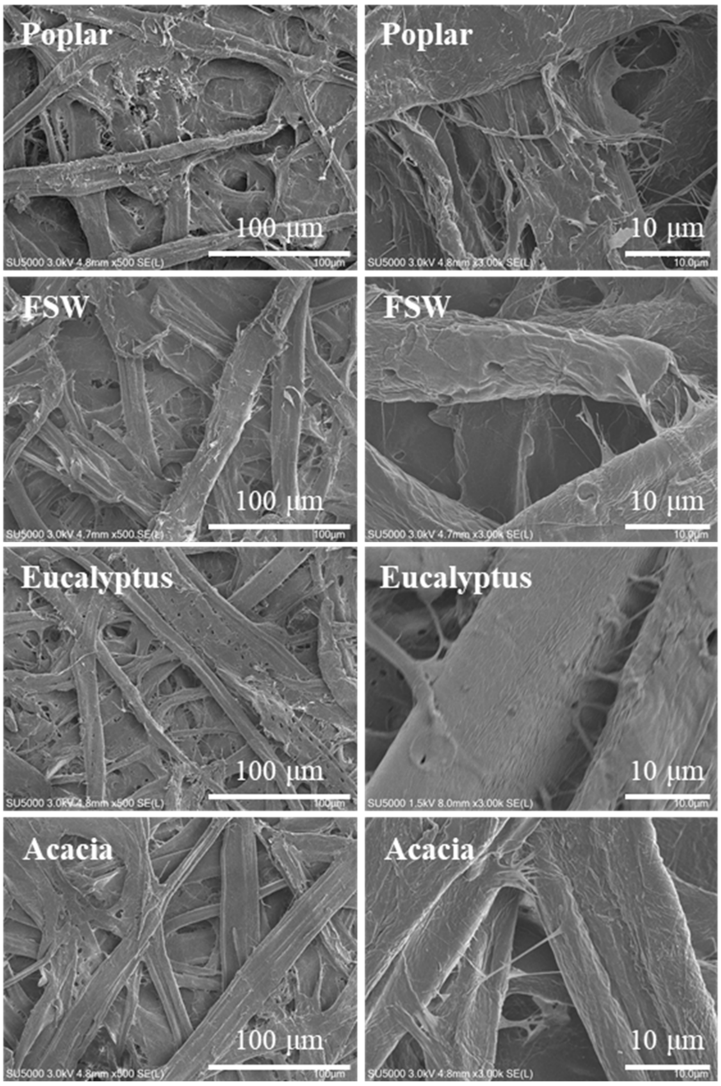

Figure S3. Differential effects of 8% NaOH concentration on the microstructure of fibers from various hardwood species
